# *Eucommia ulmoides* Oliver's Multitarget Mechanism for Treatment of Ankylosing Spondylitis: A Study Based on Network Pharmacology and Molecular Docking

**DOI:** 10.1155/2022/3699146

**Published:** 2022-10-11

**Authors:** Hong-Sheng Zhang, Sheng-Nan Zhang, Wei-Kun Guo, Sheng-Hua He

**Affiliations:** ^1^Fourth Clinical Medical College of Guangzhou University of Chinese Medicine, Shenzhen, Guangdong, China; ^2^Ruikang Hospital Affiliated to Guangxi University of Chinese Medicine, Nanning 530011, China; ^3^Department of Orthopedics, Shenzhen Traditional Chinese Medicine Hospital, Shenzhen, Guangdong, China

## Abstract

**Background:**

*Eucommia ulmoides* Oliver (EU) is a plant used in Chinese medicine as a medicinal herb to treat autoimmune and inflammatory conditions. We used network pharmacology to examine the active ingredients and estimate the main targets and pathways affected by EU when it is used to treat ankylosing spondylitis (AS).

**Materials and Methods:**

The Traditional Chinese Medicine Systems Pharmacology Database and Analysis Platform was used to search for active ingredients in EU and their target proteins. The GeneCards Database was used to find AS-related targets. The targets from the EU and AS searches that coincided were selected by constructing a Venn diagram. Then, a STRING network platform and Cytoscape software were used to analyse the protein-protein interaction (PPI) network and key targets. The strong affinity between EU and its targets was confirmed using molecular docking techniques. The Gene Ontology and the Kyoto Encyclopaedia of Genes and Genomes (KEGG) pathway enrichment analysis of overlapping targets was performed using the database for annotation, visualization, and integrated discovery online tool.

**Results:**

The number of active ingredients against AS in EU was discovered to be 28. Major targets against AS in the PPI network and core targets analyses were identified as IL-1B, PTGS2, IL-8, nMMP-9, CCL2, MYC, and IL-2. Furthermore, molecular docking studies showed the strong affinity between EU's bioactive molecules and their AS targets. Enrichment analysis revealed that active ingredients from EU were involved in a variety of biological processes, including the response to molecules derived from bacteria, extracellular stimuli, nutrient levels, and the regulation of reactive oxygen species, all of which are mediated by interleukin-17, TNF-*α*, and other signalling pathways.

**Conclusion:**

The therapy for AS using EU involves a multitarget, multipathway, and multiselection mechanism that includes anti-inflammatory and analgesic effects. This study provides a theoretical basis for future research into targeted molecular therapies for AS.

## 1. Introduction

Ankylosing spondylitis (AS) affects the spine and sacroiliac joints and is a chronic inflammatory illness. Due to the inflammatory damage induced by AS, patients have persistent back pain and morning stiffness, which limits their spinal mobility [1]. Furthermore, AS commonly aggravates extraarticular conditions such as uveitis [2]. Multiple environmental and genetic factors are known to have a role in determining the likelihood of acquiring this condition, even though the underlying pathophysiology is still unclear. More than 90% of the genetic causes have been determined so far. Research has revealed that AS is associated with the HLA-B27 gene [3]. Patients suffering from AS are frequently treated with nonsteroidal anti-inflammatory medicines, disease-modifying antirheumatic drugs, and tumour necrosis factor-alpha (TNF-*α*)-specific therapies, with surgery recommended in cases where there is severe pain or joint damage. However, long-term medication has been linked to several adverse effects, including cardiovascular, gastrointestinal, and renal concerns [4, 5]. Another significant disadvantage of this treatment is the high cost of these medications [6].

Traditional Chinese medicine (TCM) divides AS into three categories: lower back pain, spinal stiffness, and kyphosis. Many researchers have studied the function of TCM in the treatment of AS [7]. *Eucommia ulmoides* Oliver (EU), known as ‘du zhong' in Chinese and native to China, is the only plant in the *Eucommia* genus of the Eucommiaceae family. The chemical constituents of EU leaves include phenylpropanoid compounds such as sitosterol (SIT), chlorogenic acid, and ursolic acid; iridoids such as eucommiol, geniposide, geniposidic acid, and aucubin; and flavonoids such as quercetin and kaempferol [8]. As a natural herb, EU may help to strengthen bones and muscles, improve liver and kidney function, and relieve pain in the waist region and the knees [9]. It is most often used to treat renal failure and lumbago, muscular and bone weakness, and haemorrhage during pregnancy [10].

The extract of EU also lowers the activity of the PI3K/Akt pathway, which helps to reduce the production of inflammatory mediators [11]. One of the main ingredients of Jitongning tablets is EU, and these are regularly used to treat AS and rheumatoid arthritis. In a clinical trial, Jitongning tablets were shown to be an effective and safe therapy for AS [12, 13]. In addition, EU contains various phytochemicals including lignin, phenolics, terpenoids, and flavonoids [14]. However, the chemical component of EU that is effective in the treatment of AS may be unknown, so this needs further study.

Regarding biological networks, network pharmacology is a uniquely promising and cost-effective strategy for discovering bioactive components, predicting drug targets, and analysing mechanisms of drug action [15]. Furthermore, network pharmacology, in contrast to experimental pharmacology approaches, stresses multichannel control of signalling pathways, making it particularly well suited to explaining the mechanism of TCM, as this involves many chemical components and molecular targets [16].

In this research, we used network pharmacology to examine the active constituents of EU for the treatment of AS and suggest the main targets and pathways involved for the first time. The targets of EU active ingredients in the treatment of AS were analysed and their enrichment analysis was carried out. The targets were then preliminarily confirmed using molecular docking, which might provide a theoretical basis for later research into targeted molecular therapies for AS.

## 2. Materials and Methods

### 2.1. The Study Design and Workflow

**Figure 1:**
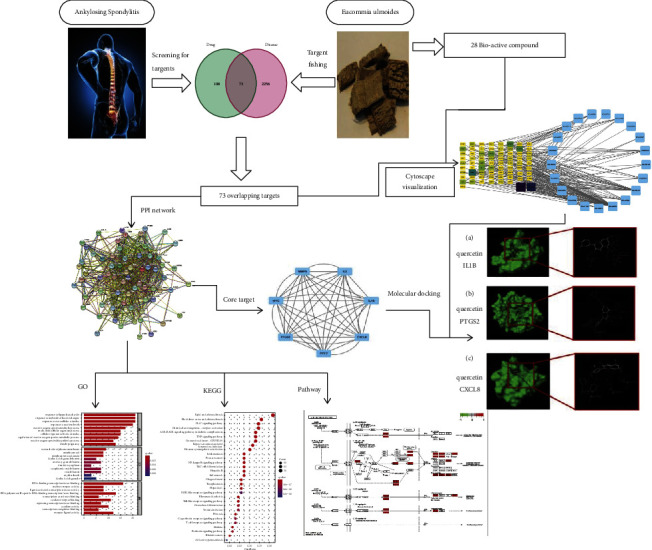
Workflow of the study design.

Figure 1 depicts the general design of this study.

### 2.2. Searching for Active Ingredients

The TCM Systems Pharmacology Database and Analysis Platform (TCMSP) (https://tcmspw.com/tcmsp) [17] was used to look for active ingredients in EU. The rate and degree to which a medicine is absorbed into the body's circulation is referred to as its oral bioavailability (OB). Drug-like features (DL) describe the functional groups or physical characteristics of a chemical compound that are the same or comparable to those of known drugs. The Caco-2 cell line from human intestinal epithelial cells is often used in studies of absorption and transportation in intestinal epithelial cells. The half-life of a drug is the critical statistic for estimating the dosing interval, dosage delivered, and drug accumulation since it indicates the drug's concentration in the blood or tissues [16]. As previously reported [18], the compounds with greater activity, OB > 30% and DL > 0.18 were further screened.

### 2.3. Searching for Drug Targets and Construction of ‘Active Drug-Target Component' Network

Drug target proteins were discovered when the active ingredients identified as described in Section 2.2 were entered into the TCMSP database for retrieval. After that, UniProt numbers were acquired. The database (https://www.uniprot.org/) was used to connect the target proteins and gene information. After entering the active ingredients and target proteins into the Cytoscape 3.8.0 program, an ‘active ingredient-target component' network was created. To select important active ingredients, a significance analysis was performed.

### 2.4. Acquisition of Potential Targets for the Treatment of AS by EU

The OMIM (https://omim.org/), PharmGKB (https://www.pharmgkb.org/), TTD (https://db.idrblab.net/ttd/), DrugBank (https://go.drugbank.com/), and GeneCards (https://www.genecards.org/) databases were used to find AS-related targets. To specify the AS condition, the terms ‘ankylosing spondylitis' or ‘AS' were used; the term ‘*Homo sapiens*' was used to search for human disease targets related to AS. Using a Venn diagram, the pharmacological targets that were identified in Section 2.2 were mapped to relevant disease targets. To discover the possible specific targets of EU in the treatment of AS, common nonspecific targets were filtered out.

### 2.5. Protein-Protein Interaction Network and Core Target Analysis

The chosen targets were entered into the STRING network platform (https://string-db.org/) [19] to create a protein-protein interaction (PPI) network to determine the probable direct targets of EU and the interactions between them. The confidence level was set to medium, and the protein type was set to *Homo sapiens* (0.400). The PPI network was built using Cytoscape 3.8.0 software, and the key targets were examined and selected.

### 2.6. Gene Ontology (GO) Annotation

The key mechanisms of the action of the targets were investigated using GO enrichment. The GO enrichment of EU's targets in treating AS was investigated using the ClueGO plug-in in Cytoscape. Only relevant GO keywords with *P* < 0.05 were presented [20], and the protein type was assigned to *Homo sapiens*. We used the ‘Analysis' function to create a graphic depiction of the top 10 elements in biological process (BP), molecular function (MF), and cellular component (CC) based on their relevance.

### 2.7. Kyoto Encyclopaedia of Genes and Genomes (KEGG) Pathway

The KEGG pathway was used to further explain the function of the targets in metabolism, signal transduction, and other activities by monitoring their distribution throughout the network. For pathway enrichment analysis, the targets were loaded into the database for annotation, visualization, and integrated discovery (DAVID) database (https://david.ncifcrf.gov/). The official gene symbols were used as the identifiers. GeneList was chosen as the list type. *Homo sapiens* was the only species available. Then, we looked for KEGG pathway information that differed significantly. We used R software, R 3.6.0 for Windows to create a bubble plot of the signalling pathways. We used the DAVID database to download the most enriched pathway, and the enriched genes were indicated with red stars.

### 2.8. Verification of Compound-Target Interaction Using Molecular Docking

To represent the relationship between small molecule ligands and protein receptors, molecular docking technology developed based on the lock and key concept. In addition, based on the conformation change and energy matching procedure, the optimal binding mode and location were found. The SYBYL-X 2.1.1 for Windows was used in this work to confirm the compound-protein target interaction. The Research Collaboratory for Structural Bioinformatics Protein Data Bank database (https://www.rcsb.org/) was used to get the crystal structures of the protein targets. The imported crystal structures were used to build the receptor protein grid in the molecular operating environment (MOE), which was related to protonation, water removal, repeated structure building, structure preparation, and energy reduction. The ligand binding site was chosen using the receptor grid design. Finally, to acquire the graphical results of molecular docking, the three-dimensional BioData Mining (2020) 13 : 12 Page 5 of 18 chemical structure was altered using ChemBioDraw software, imported into the MOE, and docked with the protein construct.

## 3. Results

### 3.1. Searching for Bioactive Compounds in EU and Their Related Targets for the Treatment of AS

The TCMSP was used to identify 28 active compounds from EU and 1474 associated targets based on the following criteria: OB > 30% and DL > 0.18 (Table 1).

### 3.2. Active Drug-Target Component Network Construction and Analysis

**Figure 2:**
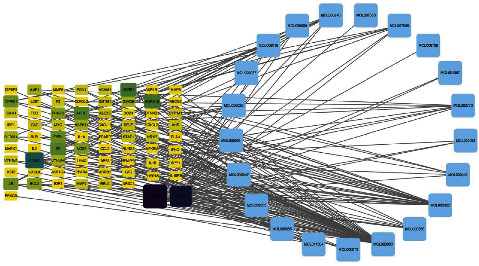
Bioactive compounds and corresponding target network of Duzhong in the treatment of AS. Yellow represents the active ingredient, blue represents the target, and each edge represents the interaction between the nodes.

The network had 784 edges and 73 nodes, including 21 active ingredient nodes and 73 target nodes (Figure 2). The network topology characteristics were analysed using the network analysis tool. We looked at the nodes that had been selected the most. These nodes were shown to have an important function in the network and might represent important chemicals or targets. The network's active components had an average selection value of 21.5. We conducted a study to determine the association between the active ingredient and AS target (Table 2).

### 3.3. Potential Targets of EU in the Treatment of AS

**Figure 3:**
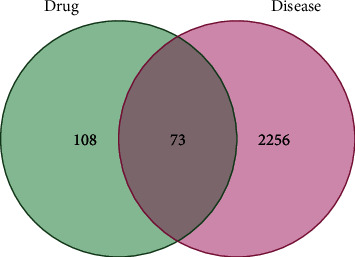
Venn diagram of Duzhong and AS targets.

The OMIM, PharmGKB, TTD, DrugBank, and GeneCards databases were used to find 2,266 AS targets. After mapping EU-related targets and AS-related targets in a Venn diagram, 73 targets overlapped with matching target-related bioactive compounds. Finally, Cytoscape was used to show 21 bioactive substances with their corresponding 73 overlapping targets. This highlights the targets of EU's active ingredients for the treatment of AS (Figure 3).

### 3.4. Construction of the PPI Network and Core Targets

**Figure 4:**
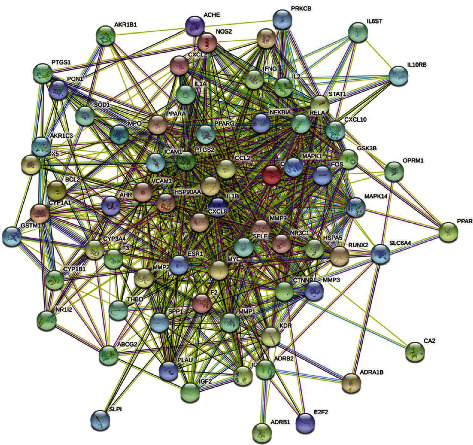
Construction of the PPI network and core targets. (a) the PPI network of potential targets of TWH in the treatment of AS. (b) Core targets of overlapping targets.

As shown in Figure 4 the network included 73 nodes and 784 edges, with an average node degree of 21.5. The size, color, and degree value of the nodes in the diagram were all positively associated. The greater the size and darker the hue of nodes, the higher their degree value. A greater degree suggested stronger interactions with other proteins, which might play a key role in the biological activity. The proteins IL-1B, PTGS2, IL-8, nMMP-9, CCL2, MYC, and IL2 were the top seven targets in terms of degree value (Figure 4), and they may be important targets for EU's effectiveness in the treatment of AS. We undertook a review of the literature to see whether there was a link between these active components and AS or inflammation (Table 3).

### 3.5. Enrichment Analysis of the GO Annotation and the KEGG Pathway

**Figure 5:**
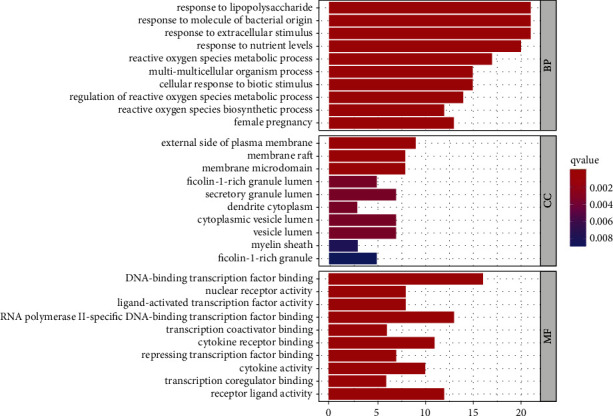
GO bar plot diagram of Duzhong in the treatment of AS.

The FunRich 3.1.3 program screened a total of 1734 GO items (*P* < 0.05). There were 1604 BP, 105 MF, and 25 CC protein targets in all. Responses to lipopolysaccharide (LPS), bacterial molecules, extracellular stimuli, nutrient levels, reactive oxygen species metabolic processes, multicellular organism, biotic stimuli, and female pregnancy are all examples of BP. DNA-binding transcription factor binding, nuclear receptor activity, ligand-activated transcription factor activity, RNA polymerase II-specificDNA-binding, transcription factor binding, transcription coactivator binding, cytokine receptor binding, and repressing transcription factor binding are all examples of MF. The exterior side of the plasma membrane, membrane raft, membrane microdomain, ficolin-1-rich granule lumen, and secretory granule lumen are all part of the CC. All the top ten protein targets are shown in Figure 5.

**Figure 6:**
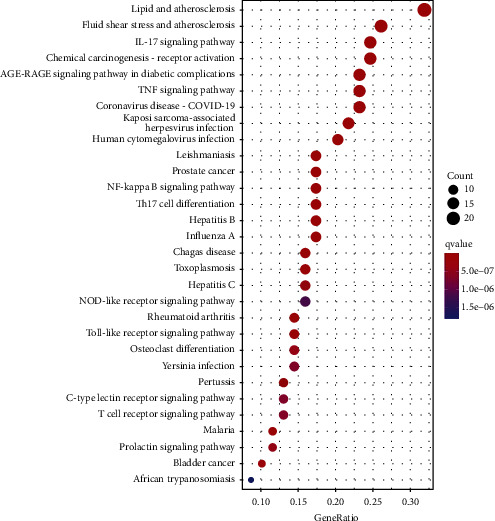
Bubbles in the KEGG pathway of AS treated by Duzhong. The vertical axis represents pathway names, and the horizontal axis represents the percentage of enrichment factors. The size and color of bubbles represent the number of enrichment targets and *P* value, respectively. The redder the color is, the smaller the *P* value is.

**Figure 7:**
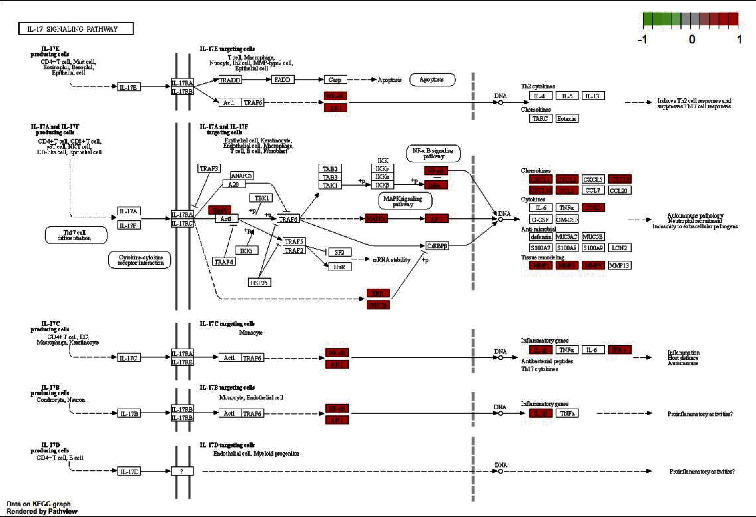
Related targets of Duzhong and IL-17 signaling pathway. Red star indicates the intersection target of drug and disease.

Response to molecules of bacterial origin, LPS, response to extracellular stimuli, response to nutrient levels, reactive oxygen species, multi-cellular organism processes, cellular response to biotic stimuli, and regulation of reactive oxygen species were among the 139 KEGG-enriched signalling pathways discovered by searching the DAVID database (*P* < 0.05).  Figure 6 depicts the top 30 signalling pathways with the greatest enrichment significance. Simultaneously, we conducted a KEGG analysis on the interleukin-17 (IL-17) signalling pathway as an example. Multiple components of the IL-17 signalling cascade, including AP1 and Hsp90, were linked to AS, according to these findings (Figure 7).

### 3.6. Verification of Compound-Target Interactions

**Figure 8:**
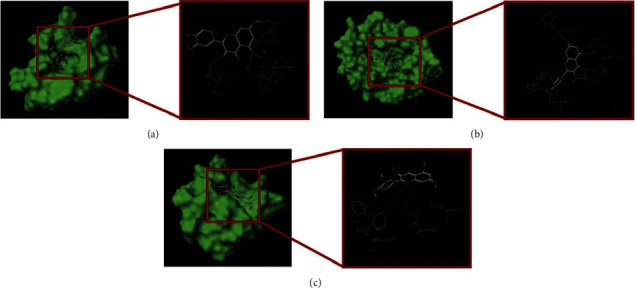
Molecular docking pattern diagram. Molecular docking diagram of quercetin and IL1B (a), of quercetin and PTGS2 (b), and (c) of quercetin and CXCL8. The yellow line represents the hydrogen bond interaction force between the two, which drives the molecule to bind to the active site.

The top three target proteins in the PPI network with intermediate value (IL-1B, PTGS2, and IL-8) were chosen, and the active ingredient quercetin was rated first in the median of the ‘active drug-target component' network. For molecular docking, SYBYL-X 2.1.1 was employed (Table 4). Higher absolute docking scores imply a greater binding force between the molecule and the target, and a more stable molecular conformation [46, 47]. A docking score absolute value > 4.25 suggests average binding activity, >5.0 shows good binding activity, and >7.0 indicates excellent binding activity. Molecular docking experiments revealed that PTGS2 had the greatest affinity and binding energy for quercetin. Each protein was effectively docked with the bioactive compound. Quercetin, extracted from the compound-target network, and a stable docking model with a particular binding location, distance, and structure was obtained. The binding site where the drug's active component interacts with the target protein may be as shown in Figures 8(a)–8(c).

## 4. Discussion

The rational method of network pharmacology is used to investigate the possible biological mechanisms of TCM in the treatment and prevention of many illnesses, particularly chronic and recurrent disorders [48]. Previous research has shown that EU has a key role in the treatment of AS [49], although the mechanism of the action is still unknown. For the first time, pharmacology was employed to comprehensively reveal the mechanism of the action of EU against AS as a network of effects. Furthermore, the compound-target stable molecular docking model demonstrated successful binding between representative compounds and key AS targets, confirming the interactions between EU active ingredients and protein targets affecting AS. These examples of computer models/figures demonstrated EU's direct influence on AS and possible methods including ‘multiple chemicals, multiple targets, and multiple mechanisms.'

Previous studies have found that many TCM components play important roles in many human diseases. For example, *Averrhoa carambola* leaf extract depresses the central nervous system in the thiopental-sodium model of Swiss albino mice [50]. Also, the leaf extract of *Lannea coromandelica* is potentially antidiabetic [51]. In addition, polyphenol nanoformulations have been used to treat diabetes mellitus [52]. As reported by Saikat Mitra et al., the leaf extract of *Avicennia alba* has antidiabetic, anti-inflammatory, analgesic, and antidiarrheal activity [53]. Furthermore, zinc oxide nanoparticles and natural small molecules can act as important regulatory factors in human cancer [54, 55]. Bioactive compounds such as resveratrol also have therapeutic effects on Alzheimer's disease [56, 57]. In this study, quercetin, kaempferol, ent-epicatechin, catechin, and beta-SIT were discovered as the most significant active ingredients of EU in the treatment of AS after searching for all the active ingredients and analysing the compound-target network. Previous research has shown that quercetin stimulates chondrocyte proliferation and acts on inflammatory damage to speed up cartilage repair [58]. Kaempferol is increasingly being linked to the inflammatory process. Kaempferol's anti-inflammatory properties include that it regulates cytokines and inhibits the MAPK pathways [59, 60]. Also, by downregulating ICAM-1 and VCAM-1, kaempferol can ameliorate endothelial dysfunction and rheumatic disease symptoms. Epicatechin has antioxidant properties, may efficiently scavenge free radicals, and inhibits the cell's oxidative potential [61]. The activity of cells involved in the immunological response is modulated by ent-epicatechin [62]. The catechin group's most significant actions include anti-inflammatory, antioxidant, and chemopreventive activity [63]. The compound SIT is a bioactive phytosterol found in plant cell membranes that has a chemical structure like cholesterol found in human cells [64].

Notably, these EU active ingredients work together to produce anti-inflammatory, anti-apoptotic, and cartilage homeostatic effects, all of which might be considered viable treatment options for AS.

The PPI network analysis revealed that IL-1B, PTGS2, IL-8, and nMMP-9 may be significant targets for EU's effectiveness in the treatment of AS. These targets had the most contact with others in EU's overall active ingredient-AS target network, and they were most likely to cause a cascade of antagonistic effects on AS. Furthermore, gene targets working on several pathways were discovered in the CTP network, indicating the relevance of certain targets in the broader AS bioinformatics network. This also supported EU's ‘multiple targets-multiple routes' regulatory mechanism.

The cytokine IL-1B is a proinflammatory cytokine generated primarily by activated macrophages and belongs to the IL-1 family. Monocytes and macrophages and B and T lymphocytes are all activated by IL-1B, which causes fibroblast proliferation and synovial pannus development. In addition, IL-1B has a role in chronic inflammation. Furthermore, the intracellular actions of IL-1A and IL-1B may cause inflammation in AS [29]. There are currently few studies linking IL-1 and AS. Chromosome location 2q13 is associated with AS. The interleukin 1 (IL-1) gene cluster is located 132 cm from the telomere of chromosome 2p, in a region linked to AS risk [30]. Prostaglandins implicated in inflammation and/or cell proliferation are produced by PTGS2. The elevation of PTGS2 by inflammatory agents and during inflammatory responses in cells that mediate inflammation, and the decrease of PTGS2 expression in the presence of anti-inflammatory drugs, provide significant evidence that this enzyme plays a role in inflammation [31]. Interleukin IL-8 is generated by fibroblasts and capillary endothelial cells in response to a variety of stimuli, including exogenous microbial products and proinflammatory cytokines including IL-1 and TNF-*α*. The mechanism of IL-8 action in AS, like that of IL-1, is unclear. The levels of IL-8 are higher in patients with AS, according to existing research [32]. In osteoarthritis, nMMP-9-mediated syndecan-4 shedding correlates with severity [33]. In addition, nMMP-9 protects against LPS-induced inflammation in osteoblasts [34]. While the above research found several EU active ingredients acting on AS targets, how do these targets affect AS? More research was needed.

Our KEGG study demonstrated that lipids and atherosclerosis, the IL-17 signalling pathway, and the TNF signalling pathway are all linked to EU's therapeutic mechanism for the treatment of AS. As a chronic inflammatory disease, AS affects the spine and sacroiliac joints. It has the potential to harm the skeletal system's structural and functional integrity. Several other organs, including the eyes, skin, gastrointestinal tract, and cardiovascular system (CVS) may also be damaged [65]. Among CVS disorders, atherosclerosis is one of the leading causes of morbidity and mortality [66]. The major risk factor for atherosclerosis is dyslipidaemia [67]. The abnormalities in lipid profiles in people with AS are assumed to be caused by systemic inflammation [68]. Systemic inflammation triggers the generation of free oxygen radicals via immune-mediated pathways, which have been linked to the pathophysiology of inflammatory diseases. The formation and progression of atherosclerosis are influenced by systemic inflammation, endothelial inflammation, and increased oxidative stress [69]. Several clinical investigations [70] have shown that TNF-*α*, coupled with IL-17 and interleukin-23 (IL-23), plays a crucial role. The cytokine IL-17 has been demonstrated to improve T cell priming and induce the production of proinflammatory mediators (such as IL-1, IL-6, TNF-*α*, and chemokines) by a variety of cell types, including fibroblasts, endothelial cells, macrophages, and epithelial cells [71]. The cytokine IL-17 has also been found to play a key role in the effector phase of the inflammatory response. The expression of IL-17 is elevated in AS patients [72]. The signalling mechanisms involving TNF-*α* are complicated and not completely understood. When TNF-*α* binds to the TNFR1, it activates proinflammatory and programmed cell death pathways, which are linked to tissue damage, according to results from *in vitro* and *in vivo* studies. The TNFR2 has been demonstrated to modulate tissue healing and angiogenesis signals [73]. The signalling molecule TNF-*α* has been found in the sacroiliac joints of individuals suffering from AS, especially in the early active stages of the condition [74, 75], and increased blood TNF-*α* levels are linked to the disease activity.

Molecular docking experiments also revealed that a representative selection of AS targets, IL-1B, PTGS2, and IL-8 had a high affinity for the active ingredients of EU. In this research, network pharmacology was employed to build on EU's possible anti-AS actions, which were then visualised using molecular docking. However, the findings from the prediction need additional experimental verification due to the limits of network pharmacology and molecular docking technologies.

## 5. Conclusion

According to this research, treatment of AS using EU might involve a complex multitarget, multipathway, and multiselection mechanism. The proteins IL-1B, PTGS2, IL-8, nMMP-9, CCL2, MYC, and IL2 were identified as major targets against AS in the PPI network and core target analyses. The strong affinity between EU's bioactive molecules and their AS targets was confirmed. As a medicinal herb, EU may be used as an analgesic, immunomodulator, antimicrobial, and anti-inflammatory. This study might provide a theoretical basis for research into targeted molecular therapies for AS.

## Figures and Tables

**Table 1:** The chemical formula and chemical structure of key active components.

Numbers	Molid	Molecule names	Chemical
1	MOL002058	40957-99-1	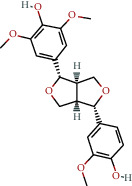

2	MOL000211	Mairin	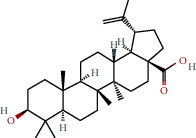

3	MOL000358	Beta-sitosterol	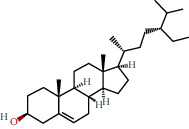

4	MOL000422	Kaempferol	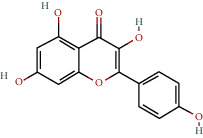

5	MOL004367	Olivil	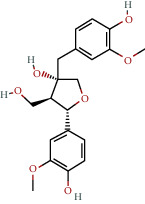

6	MOL000443	Erythraline	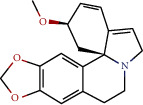

7	MOL005922	Acanthoside B	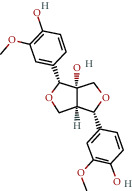

8	MOL006709	AIDS214634	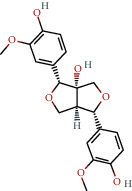

9	MOL007059	3-Beta-hydroxymethyllenetanshiquinone	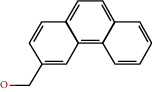

10	MOL000073	Ent-epicatechin	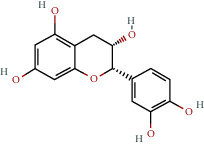 0

11	MOL007563	Yangambin	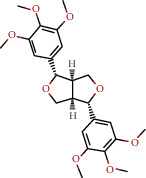 1

12	MOL009007	Eucommin A	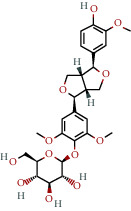

13	MOL009009	(+)-medioresinol	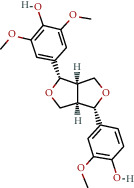

14	MOL009015	(−)-Tabernemontanine	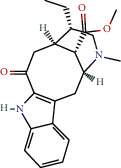

15	MOL009027	Cyclopamine	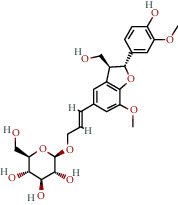

16	MOL009029	Dehydrodiconiferyl alcohol 4, gamma'-di-O-beta-D-glucopyanoside_qt	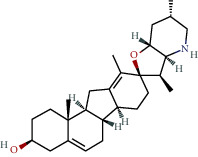

17	MOL009030	Dehydrodieugenol	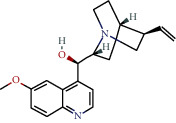

18	MOL009031	Cinchonan-9-al, 6′-methoxy-, (9R)-	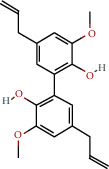

19	MOL009038	GBGB	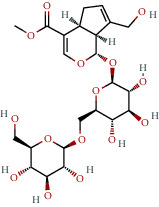

20	MOL009042	Helenalin	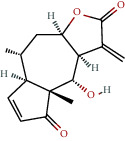

21	MOL009047	(+)-eudesmin	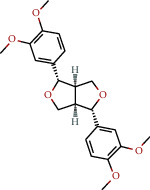

22	MOL009053	4-[(2S, 3 R)-5-[(E)-3-hydroxyprop-1-enyl]-7-methoxy-3-methylol-2,3-dihydrobenzofuran-2-yl]-2-methoxy-phenol	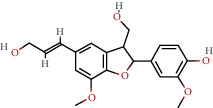

23	MOL009055	hirsutin_qt	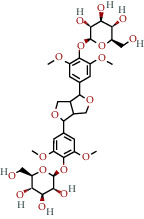

24	MOL009057	liriodendrin_qt	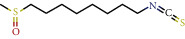

25	MOL000098	Quercetin	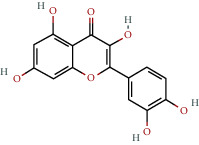

26	MOL002773	Beta-carotene	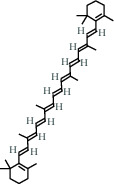

27	MOL008240	(E)-3-[4-[(1R,2 R)-2-hydroxy-2-(4-hydroxy-3-methoxy-phenyl)-1-methylol-ethoxy]-3-methoxy-phenyl]acrolein	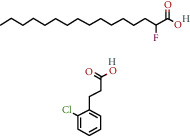

28	MOL011604	Syringetin	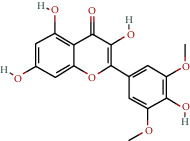

**Table 2:** Analysis on the relationship between active ingredients and AS.

Numbers	Active ingredient accession numbers	Names	Description	References
1	MOL007536	PTGS2	PTGS2 high expression in patients with AS	[21, 22]
2	MOL007059	PTGS1	PTGS1 and AS have a chain relationship in genetics	[23]
3	MOL006709	HSP90AA1	At present, there is no mechanism study, but it can be used as the target of Chinese traditional medicine yun-pi-yi-shen-tong-du-tang to treat AS.	[24]
4	MOL004367	ADRA1B	No available data	
5	MOL002772	ADRB2	No available data	
6	MOL002058	SLC6A4	No available data	
7	MOL000443	OPRM1	No available data	
8	MOL000422	BCL2	No available data	
9	MOL000358	PON1	PON1 activity was significantly associated with AS, that is, people with higher AS activity were less likely to get sick.	[25]
10	MOL000098	NOS2	SNP rs2297518 of NOS2 gene was significantly associated with AS	[26]
11	MOL000073	AR	No available data	
12	MOL011604	PPARG	No available data	
13	MOL009055	F2	No available data	
14	MOL009053	ACHE	No available data	
15	MOL009057	RELA	No available data	
16	MOL009031	MMP1	AS can promote the expression of MMP1	[27, 28]
17	MOL009029	STAT1	No available data	
18	MOL009027	CYP3A4	No available data	
19	MOL009015	CYP1A1	No available data	
20	MOL009009	ICAM1	No available data	
21	MOL008240	SELE	No available data	

**Table 3:** Analysis on the relationship between key targets and AS.

Names	The role of key targets in antiosteoarthritis or anti-inflammatory and related mechanisms	References
IL1B	IL-1B belongs to the IL-1 family and is a proinflammatory cytokine mainly produced by activated macrophages. It is positively correlated with inflammation	[29, 30]

PTGS2	Inflammation regulates the expression of PTGS2. Numerous investigations have demonstrated that inflammation can express PTGS2 opportunistically.	[31]

CXCL8	CXCL8 is a chemokine that binds to the CXCR1 and CXCR2 receptors. Extensive experimental evidence indicates that CXCL8 and its receptors not only aid in pathogen elimination, but also significantly contribute to disease-related processes such as tissue damage, fibrosis, angiogenesis, and carcinogenesis.	[32]

MMP9	MMP9 and other MMP protein may be involved in the interaction between inflammatory and progenitor cells of the skeletal system. MMP9 inhibits the inflammatory response between bone cells.	[33, 34]

CCL2	CCL2 is implicated in malignant tumour metastasis, which promotes malignant tumours. In addition, CCL2 can exacerbate stomach inflammatory symptoms.	[35–38]

c-MYC	c-MYC can inhibit inflammation by activating the expression of inflammatory cytokines.	[39–43]

IL2	IL2 may indirectly promote resistance to inflammation	[44, 45]

**Table 4:** Molecular docking results of key active components and core targets of Eucommia ulmoides.

Target proteins	Structures	Active ingredients	Docking scores
IL1B	5r8q	Quercetin	5.0924
PTGS2	5ikr	Quercetin	8.4329
CXCL8	6WZM	Quercetin	3.3164

## Data Availability

All data generated or analysed during this study are included in this article.
